# Tunable actuation behavior of ionic polymer metal composite utilizing carboxylated carbon nanotube-doped Nafion matrix†

**DOI:** 10.1039/c7ra11498b

**Published:** 2018-01-15

**Authors:** Jie Ru, Zicai Zhu, Yanjie Wang, Hualing Chen, Dichen Li

**Affiliations:** State Key Laboratory for Strength and Vibration of Mechanical Structures, Xi'an Jiaotong University Xi'an Shaanxi 710049 People's Republic of China hlchen@mail.xjtu.edu.cn; School of Mechanical Engineering, Xi'an Jiaotong University Xi'an Shaanxi 710049 People's Republic of China; School of Mechanical and Electrical Engineering, Hohai University Changzhou 213022 People's Republic of China; State Key Laboratory for Manufacturing Systems Engineering, Xi'an Jiaotong University Xi'an 710049 People's Republic of China

## Abstract

In this study, we propose to neutralize the relaxation deformation of Nafion-ionic polymer metal composite (IPMC) by slow anode deformation of Flemion-IPMC caused by carboxyl groups (–COOH). Carboxylated carbon nanotubes (CCNT) as –COOH carriers were doped into a Nafion matrix. By adjusting the doping content from 0 wt% to 10 wt%, an IPMC with constant steady-state deformation has been achieved at a critical CCNT content of 2 wt%. Moreover, the increasing rate of the slow anode deformation with the CCNT content is tunable, which is found to be 2.26 mm s^−1^ %^−1^.

## Abstract 2

In this study, we propose to neutralize the relaxation deformation of Nafion-ionic polymer metal composite (IPMC) by slow anode deformation of Flemion-IPMC caused by carboxyl groups (–COOH).

## Graphical abstract

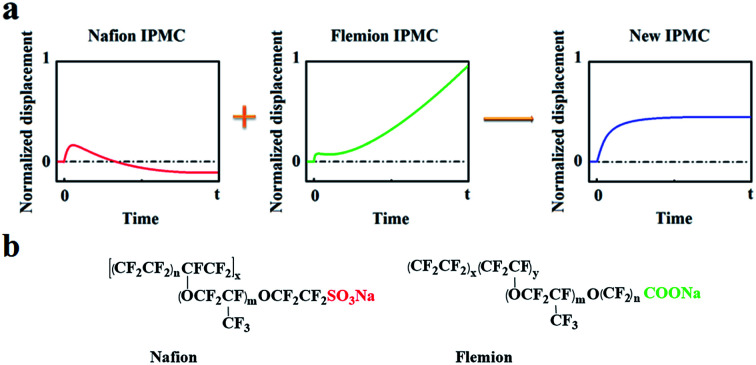

During the past two decades, ionic electroactive polymer (iEAP) actuators have been extensively studied as promising smart materials of academic interest and for industrial applications.^1–4^ As a kind of typical iEAP material, ionic polymer metal composite (IPMC) is well known to be an innovative material with great potential applications in microrobots,^5^ space exploration,^6^ and the medical field,^7^ because of its advantageous properties, such as low mass, softness, and large deformation under a relatively low driving voltage (like 1–3 V).^8,9^ In addition, as a well-known “artificial muscle”,^10^ IPMC can accomplish some unusual tasks such as “swimming” like a fish and “flying” like a bird,^11,12^ indicating enormous potential in the bionic engineering field.

However, to our knowledge, IPMC has not been widely used in practice, which is mainly due to its highly unstable deformation properties. When subjected to a DC voltage under saturated conditions, an IPMC often shows a complex unstable deformation. Actuation of Nafion (perfluorosulfonic acid polymer)-IPMC is often prone to a relaxation deformation following a fast anode deformation. Various relaxation deformations have been reported. All of Pt-, Pd-, Au-, Cu-, and Ni-Nafion IPMCs show obvious relaxation deformation,^13–16^ although there may be differences in magnitude of the relaxation of the various mentioned electrode-based IPMCs. In contrast to the Nafion-IPMC, Flemion (perfluorocarboxylic acid polymer)-IPMC still slowly bends toward the anode side after a fast initial deformation and slight relaxation.^17^ Both of the deformations are too complex to predict or to control. The currently available IPMCs cannot meet the requirements of precise control fields, such as position control, shape control and constant force drive. Hence, a new kind of IPMC performing a steady and controllable deformation is urgently required for practical engineering applications.

Based on the deformation characteristics of Nafion- and Flemion-IPMC, if the relaxation deformation of Nafion-IPMC can be neutralized adequately by the slow anode deformation of Flemion-IPMC, a constant steady-state deformation without relaxation can be obtained approximately (as shown in Fig. 1). According to such a hypothesis, a direct method can be deduced to produce a non-relaxation IPMC by utilizing a hybrid membrane with an appropriate proportion of Nafion and Flemion.

**Fig. 1:**
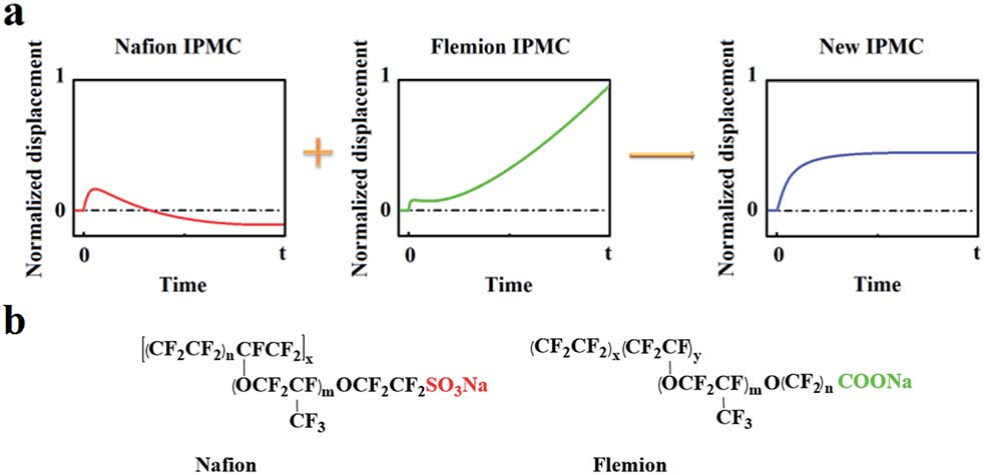
(a) Illustration of the hypothesis of neutralizing the relaxation deformation of Nafion-IPMC by the slow anode deformation of Flemion-IPMC. (b) Molecular formulas of Nafion and Flemion.

Further solution was found from the essence of relaxation and slow anode deformation. To our knowledge, Nafion is a perfluorosulfonic acid ionomer with strong acid groups (–SO_3_H) bound to the side chains. Fast anode deformation is induced by the swelling effect due to the aggregation of cations together with bonded water molecules on the cathode side, while relaxation deformation is caused by the back migration of free water to the anode side.^17–20^ In contrast, Flemion is a perfluorocarboxylic acid ionomer with weak acid groups (–COOH) bound to the side chains. For Flemion-IPMC, there is always a considerable amount of undissociated –COOH groups in the matrix after cation exchange.^21^ The slow anode deformation of Flemion-IPMC is caused by the migration of the secondary dissociated H^+^ together with bonded water molecules to the cathode side.^21^ The difference in deformation properties is strongly influenced by the quantity and direction of the movable species,^22–25^ especially the secondary dissociated H^+^. Therefore, we propose an alternative method to produce a non-relaxation IPMC by utilizing a Nafion matrix doped with –COOH groups, which can generate an amount of secondary dissociated H^+^.

Carboxylated multi-walled carbon nanotubes (CCNT), with –COOH groups covalently bonded to the surface of MWCNT and very weak van der Waals force among the bundles, are water-soluble and have numerous ion insertion sites.^26–28^ In this research, CCNT (purchased from the Chinese Academy of Sciences Chengdu Organic Chemical Co. Ltd) were doped into a Nafion matrix as –COOH carriers to confirm the above assumption. The critical CCNT content is also evaluated at which the relaxation deformation and the slow anode deformation are eliminated. Here, Pd-electrode IPMCs are employed as specimens and fabricated by assembling CCNT-doped Nafion membranes and Pd electrode layers *via* an electroless plating method.^29^ The preparation process is described in detail in the ESI.†

SEM micrographs of all the prepared Nafion membranes were obtained using a field emission scanning electron microscope (SEM, Zeiss Genimi SEM 500) and are shown in Fig. 2 in the ESI,† from which the CCNT can be seen uniformly dispersed in the Nafion matrix.

In testing, a specimen (with a certain size of 35 mm in length, 5 mm in width, (190 ± 10) μm in thickness) was clamped by a gold clamp on one end with a free length of 30 mm after wiping off the surface water with filter paper. Then a 2 V DC voltage was applied to the specimen by an arbitrary power supply (HM8143) using Labview software. The deformation was measured and recorded for 50 seconds with a laser displacement sensor (Keyence LK-G80) at the measuring point which was 20 mm from the fixed end and at ambient temperature and humidity. The test platform is shown in Fig. 2. Three parallel samples of each kind IPMC were tested, and the relative standard deviations of the displacement of each kind IPMC were no more than 20%. The results are shown in Fig. 3 in the ESI.†

**Fig. 2:**
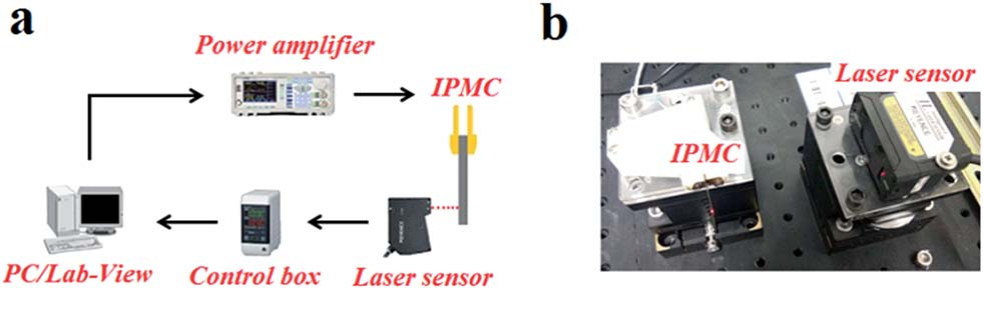
(a) Schematic of the experimental set-up. (b) Visual representation of the platform.

The time–displacement curves of the Nafion-IPMCs with various CCNT contents are shown in Fig. 3. The interesting observations from these curves are the relaxation and slow anode deformation phenomena. The 0 wt% CCNT content IPMC shows a large negative relaxation deformation (more than 2 mm), while the 1 wt% CCNT content IPMC shows a much smaller relaxation deformation (no more than 1 mm) with a tendency of straightening back rapidly. Notably, the 2 wt% CCNT content IPMC exhibits a steady anode deformation of 2.2 mm without any relaxation or slow anode deformation. With further increase in CCNT content (5 wt% and 10 wt%), the anode deformation of the corresponding IPMC becomes larger and larger, and increases infinitely during the testing period, which is very similar to the behavior of the Flemion-IPMC. The 10 wt% CCNT content IPMC exhibits the largest anode deformation of 15.4 mm, which is 7.0 times larger than that of the 2 wt% CCNT content IPMC. It is quite obvious that the deformation property is highly correlated with the content of CCNT in the hybrid membranes. As the content of the CCNT increases from 0 wt% to 10 wt%, the deformation changes from a large negative relaxation to a positive increasing deformation.

**Fig. 3:**
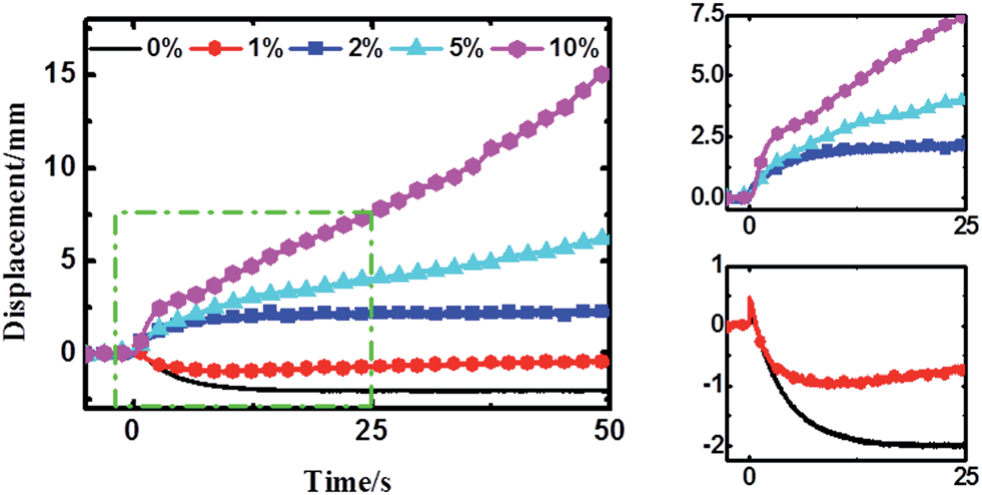
Time-displacement (at the measuring point) curves of various CCNT content Nafion-IPMCs under 2 V DC voltage. The zoomed-in regions of the green rectangle are shown in the two right-hand panels to see these changes more clearly.

The experimental results verified our hypothesis well. In general, the CCNT-doped Nafion-IPMCs show a coupling deformation behavior of pure Nafion- and Flemion-IPMC. With CCNT being doped into the Nafion matrix, –COOH groups are brought into the hybrid IPMCs (except for 0 wt% CCNT content), existing with –SO_3_H groups. Identical to Flemion-IPMCs, there exist two kinds of driving cations: Na^+^ and H^+^ in the matrix. The dissociation equations are as follows:Nafion–SO_3_Na → Nafion–SO_3_^−^ + Na^+^CNT–COONa → CNT–COO^−^ + Na^+^CNT–COOH ⇌ CNT–COO^−^ + H^+^

According to previous reports,^21^ after initial fast anode deformation, the free water molecules are driven back to the anode side because of the water concentration gradient and the pressure gradient causes the relaxation deformation, while the migration of the H^+^ cations and associated water molecules to the cathode side contributes to the slow anode deformation. Thus the CCNT/Nafion-IPMC exhibits a coupling behavior of the pure Nafion- and the Flemion-IPMC. The deformation property is highly correlated with the content of the CCNT in the hybrid membranes. The deformation varies from a large negative one to a positive increasing one when the content of CCNT changes from 0 wt% to 10 wt%. For the 0 wt% CCNT content IPMC, the fast anode deformation is induced by the aggregation of cations together with bound water molecules on the cathode side while the relaxation deformation is caused by the reversed diffusion of free water, which has been extensively studied and explained.^16–22^ The 1 wt% CCNT content IPMC still shows a negative relaxation deformation, but the amplitude of which decreases dramatically with a tendency reverting to the initial state when compared to that of the 0 wt% CCNT content IPMC. This is due to the migration of the secondary dissociated H^+^ and the carried water molecules to the cathode side (as shown in Fig. 4b). However, the amount of the secondary dissociated H^+^ is insufficient. The swelling effect of the hydrated Na^+^ and H^+^ cannot match that of the back migration of the free water. As the CCNT content increases to 2 wt%, the amount of the secondary dissociated H^+^ and the carried water molecules increases (as shown in Fig. 4c). The swelling effect of hydrated Na^+^ and H^+^ can match that of the back migration of the free water. Herein, the 2 wt% CCNT-doped IPMC shows a steady-state deformation without any relaxation deformation or slow anode deformation. With a further increase in CCNT content (5 wt% and 10 wt%), the deformation of the corresponding IPMCs, very similar to that of the Flemion-IPMC, increases infinitely. This is just because of the dramatically increased amount of the secondary dissociated H^+^ and carried water molecules (as shown in Fig. 4d). The swelling effect of the hydrated Na^+^ and H^+^ overwhelmingly offsets that caused by the back migration of the free water. As a result, the deformation property can be well controlled by doping CCNT into the Nafion matrix.

**Fig. 4:**
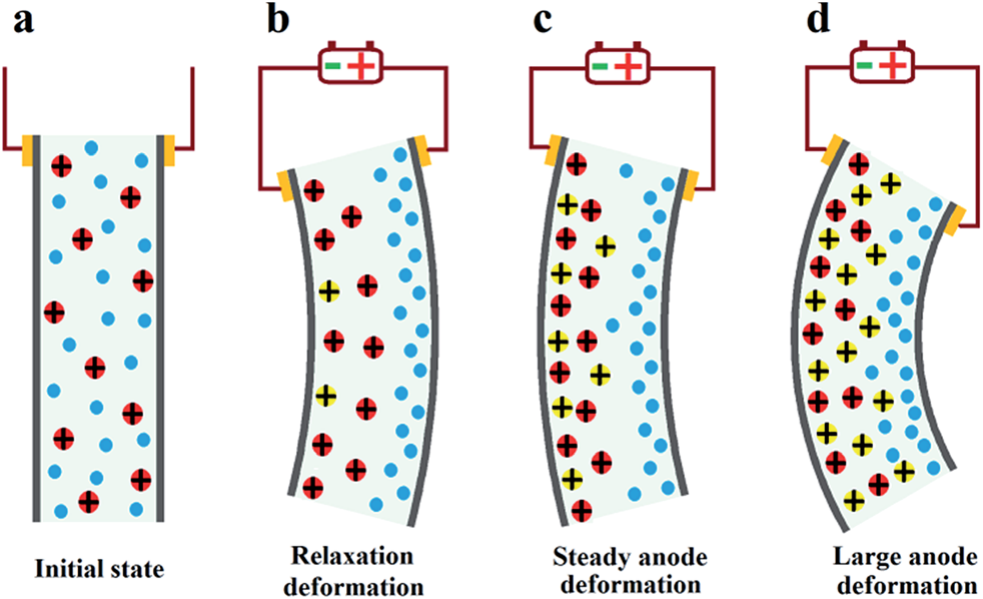
Illustration of actuation mechanism of the deformation of the IPMCs with various CCNT contents. (a) The initial state without voltage. (b) CCNT content is 0 wt% or 1 wt%. (c) CCNT content is 2 wt%. (d) CCNT content is 5 wt% or 10 wt% (blue particles: free water molecules; red particles: hydrated Na^+^ cations; yellow particles: hydrated H^+^ cations).

To further analyze the doping effect of CCNT, the deformations caused by CCNT were obtained approximately by subtracting the deformation of IPMC without CCNT doping from the deformations of IPMC with various CCNT contents. The results are shown in Fig. 5a. The deformations caused by CCNT mainly contain two parts: initial fast anode deformation and slow anode deformation, whereas the slight relaxation deformation can be ignored here. Therefore, the total deformation *d*_T_ can be described by the following equation^21^ with two diffusion processes theoretically:

1 *d*_T_ = *A*_1_(1 − e^−*t*/*τ*_1_^) + *A*_2_(1 − e^−*t*/*τ*_2_^) ≈ *A*_1_(1 − e^−*t*/*τ*_1_^) + *kt*

where the first term describes the fast anode deformation caused by the migration of hydrated Na^+^ and H^+^ cations ionized from –COONa and –COOH groups, and the second term describes the slow anode deformation caused by the migration of secondary dissociated H^+^ cations to the cathode side. *A*_1_, *A*_2_ are the amplitudes and *τ*_1_, *τ*_2_ are the characteristic times of the fast anode deformation and slow anode deformation, respectively. Usually the second dissociation of H^+^ cations is very slow, and the time constant *τ*_2_ is very large, so the slow anode deformations approximate a straight line as shown in Fig. 5a. Here, the second term can be simplified as a linear term *kt* in a limited range of time approximately. Using eqn (1) to fit the deformations in Fig. 5a, the fitting results are shown in Fig. 5b (fast anode deformation) and Fig. 5c (slow anode deformation), and the fitting effects are shown in Fig. 4 in the ESI.†

**Fig. 5:**
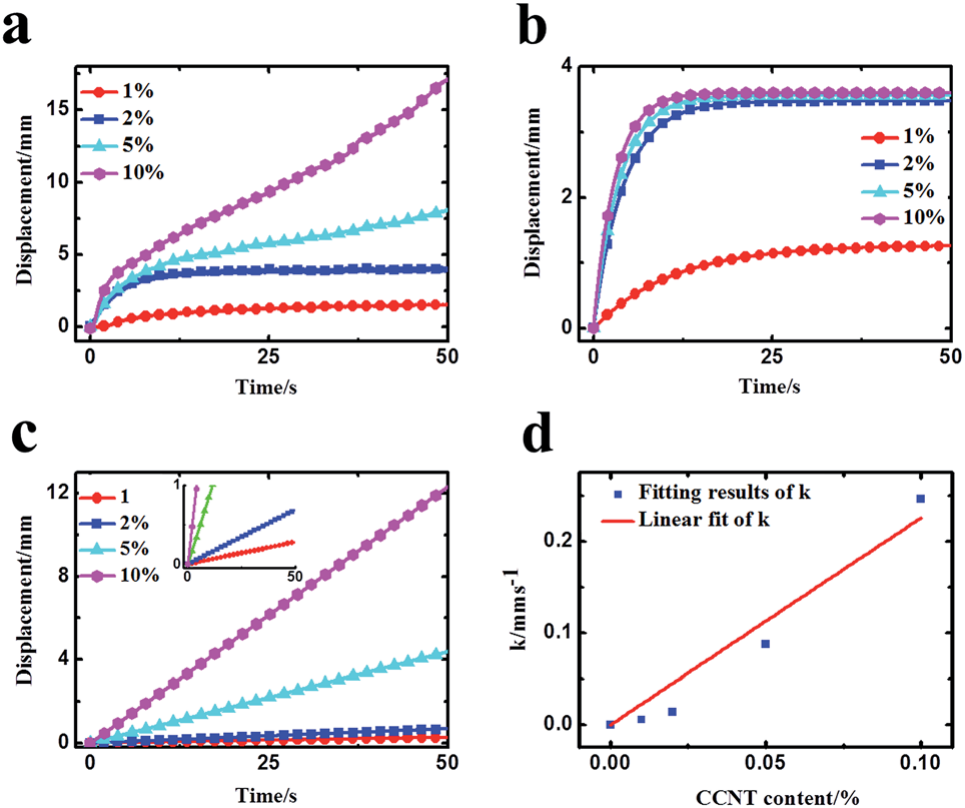
(a) The deformations caused by CCNT (obtained by subtracting the deformation of IPMC without CCNT doping from the deformations of IPMC with various CCNT contents). (b) The fast anode deformation. (c) The slow anode deformation. (d) Linear fit of growth rate (*k*) with CCNT content.

As can be seen from Fig. 5b, the fast anode deformation increases dramatically as the CCNT content increases, which is attributed to the increasing concentration hydrated Na^+^ and H^+^ cations. However, the increasing rate of the amplitude of the fast anode deformation gets smaller. To our knowledge, the hydrophilic –COOH groups give the CCNT excellent hydrophilic nature, and then form larger hydrophilic ionic clusters within the hybrid membranes.^26,28–30^ Thus the hybrid membranes can absorb a greater amount of water compared to pure Nafion membrane. This would result in an increase in the amount of free water of reverse osmosis, the swelling effect of which could offset part of that of the hydrated Na^+^ and H^+^ to a certain degree. As a result, the fast anode deformation would not increase indefinitely, and the increasing rate of the amplitude would get smaller as the CCNT content further increases. In Fig. 5c, the slow anode deformation increases significantly as the CCNT content increases from 1 wt% to 10 wt%. This is due to the dramatically increasing amount of the second dissociation of H^+^ cations from the unionized –COOH with respect to the increasing CCNT content. Fig. 5d shows the linear fit of the growth rate (*k*) with CCNT content, which indicates a direct corresponding relationship between the growth rate and CCNT content. Once the CCNT content increases to 1 wt%, the growth rate will increase by 2.26 mm s^−1^. Such results fit well with the experimental results and reveal the essence of the deformation process of the CCNT-doped IPMCs in detail, which can be used to design IPMC devices with tunable actuation behaviors for precise control fields.

## Conclusions

In summary, based on the nature of Nafion and Flemion matrices, and the deformation properties of the corresponding IPMCs, we dope CCNT as –COOH carriers into Nafion matrix to develop a new kind of IPMC performing a constant steady-state deformation. It is found that the deformation properties of the CCNT/Nafion-IPMCs are highly correlated with the content of the CCNT in the hybrid membranes. As the content of the CCNT increases from 0 wt% to 10 wt%, the deformation changes from a large negative one to a slow positive increasing one. The 2 wt% CCNT-doped IPMC exhibits a steady anode deformation without any relaxation deformation or slow anode deformation. Using a multi-diffusion equation to fit the deformations caused by the doping with CCNT, the fitting results fit well with the experimental results and can reveal the essence of the deformation process of the CCNT-doped IPMCs in detail. The increasing rate of the slow anode deformation with the CCNT content is about 2.26 mm s^−1^ %^−1^. Therefore, such results are of great significance for the design of IPMCs with tunable actuation behaviors to meet the requirements of precise control fields.

## Conflicts of interest

There are no conflicts to declare.

## Supplementary Material

RA-008-C7RA11498B-s001
