# Supplementary material for: Tunable actuation behavior of ionic polymer metal composite utilizing carboxylated carbon nanotube-doped Nafion matrix
Source: RSC Adv. 2018 Jan 15;8(6):3090–4. doi: 10.1039/c7ra11498b (PMC9077514; doi:10.1039/c7ra11498b)
Supplement: RA-008-C7RA11498B-s001 [file RA-008-C7RA11498B-s001.pdf]

Electronic Supplementary Material (ESI) for RSC Advances.
This journal is © The Royal Society of Chemistry 2017

RSC Advances

Supporting Information

1 Preparation of CCNT/Nafion membrane

Materials: The perfluorosulfonic acid (PFSA) resin Nafion solution (DE-520, 5.0-5.4 wt% in a mixture of propanol and water) was purchased from DuPont Company. Carboxylated carbon nanotubes (CCNT, purity > 98%) were purchased from the Chinese Academy of Sciences Chengdu Organic Chemical Co. Ltd. $Pd(NH_3)_4Cl_2$ (purity > 99%), $NaBH_4$ (purity ⩾ 98%), and $N_2H_4 \cdot H_2O$ (purity > 98%) were purchased from Aldrich. All of the materials were analytically pure and used without further purification.

A certain amount of CCNT was added to 3 mL ethylene glycol (EG) and sonicated for 30 min, then the suspensions was mixed with the Nafion solution and stirred for 4 hrs in a poly(dimethylsiloxane) (PDMS, Sylgad184) container (40 mm × 60 mm × 40 mm). The mixture was evaporated at 90 °C and 100 °C for 12 hrs successively to cast CCNT /Nafion hybrid membrane. After that, the membrane was annealed at 120 °C for 1 hr. The hybrid membranes were obtained with CCNT weight fraction of 1, 2, 5 and 10 wt%, respectively. The pure Nafion membrane with 0 wt% CCNT content was prepared for comparison using the same method. The sizes of membranes were 60 mm × 40 mm × (180±10) μm (length × width × thickness) under dry state. The composition of the casting solutions are summarized in table 1.

Table 1. Composition of the casting solutions.

CCNT content	Solution composition		
	CNT (mg)	Nafion(aq) (g)	EG (mL)
0 wt%	0	18.00	3.0
1 wt%	9.0	17.82	3.0
2 wt%	18.0	17.64	3.0
5 wt%	45.0	17.10	3.0
10 wt%	90.0	16.20	3.0

2 Preparation of Pd-electrode IPMCs

The major steps of the preparation process for the IPMC are as follows: 1) Surface roughening treatment. The membranes were roughened with a sandblasting machine, then washed with 20% ethanol solution in an ultrasonic cleaning machine and boiled in 1 M HCl and water for 30 min, successively. 2) Ion adsorption. This step was to soak the membranes in $Pd(NH_3)_4Cl_2$ solution to adsorb $[Pd(NH_3)_4]^{2+}$ via an ion-exchange process. 3) Reduction. In this step, the adsorbed $[Pd(NH_3)_4]^{2+}$ was reduced to metallic state by strong reducing agent $NaBH_4$ to form infiltrate electrode. 4) Further plating. In order to increase the thickness of the surface electrode and reduce the surface resistivity effectively, $Pd(NH_3)_4Cl_2$ and H_2NNH_2 were put into the same solution simultaneously to grow Pd nano-particles above the infiltrate Pd electrode layer. 5) Ion exchange. In this step, the IPMCs were cut into specimens with a certain size (35-mm long, 5-mm wide, (190±10)-μm thick) and soaked in 2 mol/L sodium hydrate solution at room temperature for 6 hrs to exchange H^+ for Na^+ as actuating cations.

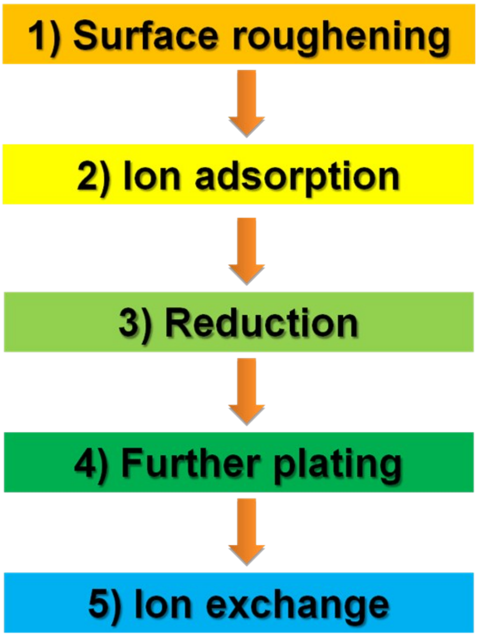

FIG. 1. The major steps of the preparation process for the IPMC.

This journal is © The Royal Society of Chemistry 20xx

3 SEM micrographs

SEM micrographs of all the prepared Nafion membranes are observed using a field emission (FE)-scanning electron micrography (FE-SEM, Zeiss Genimi SEM 500) and shown in Fig. 2, from which the CCNT can be seen uniformly dispersed in Nafion matrix.

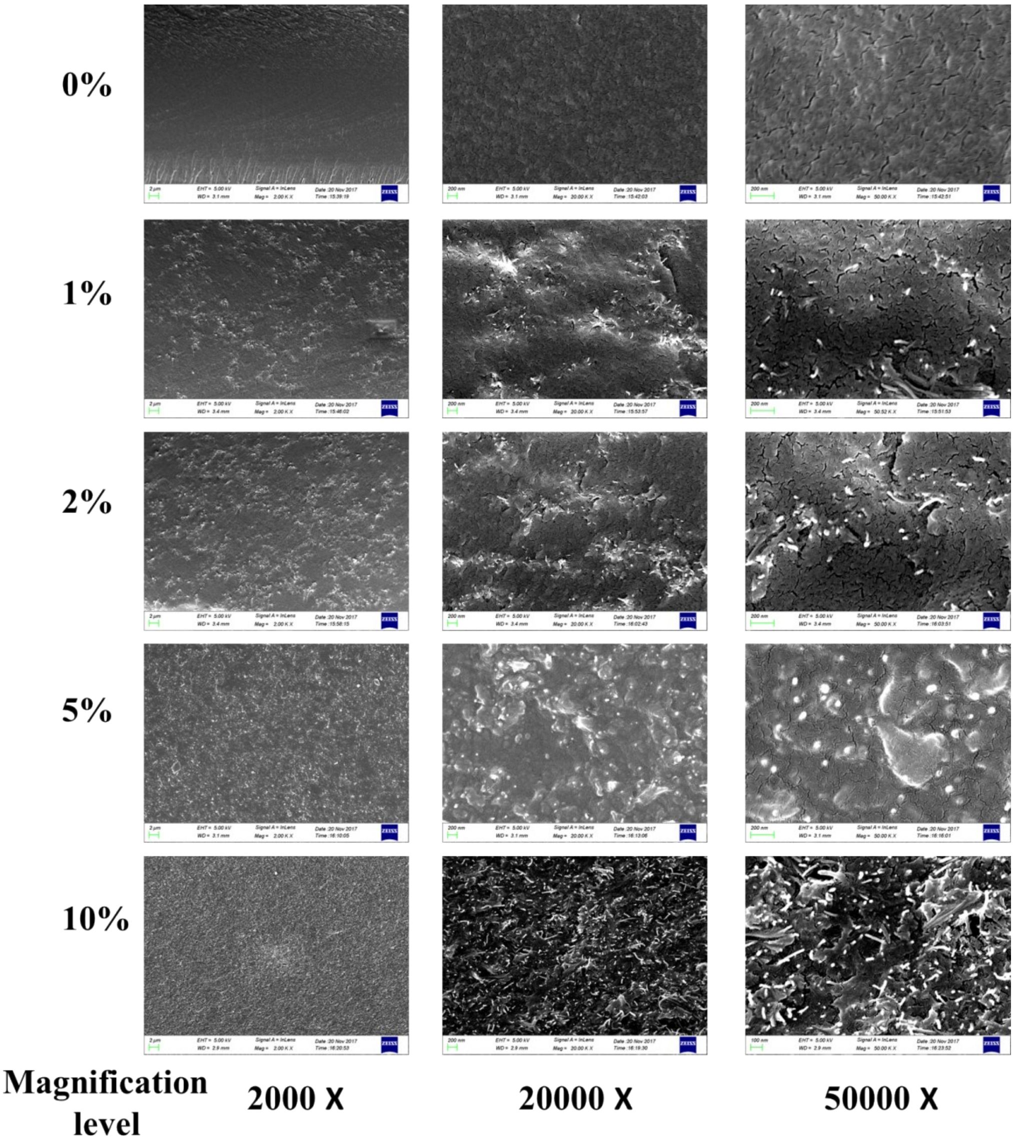

FIG. 2. SEM micrographs of the prepared Nafion membranes.

This journal is © The Royal Society of Chemistry 20xx

Please do not adjust margins

4 The measured displacement of three parallel samples of each kind IPMC

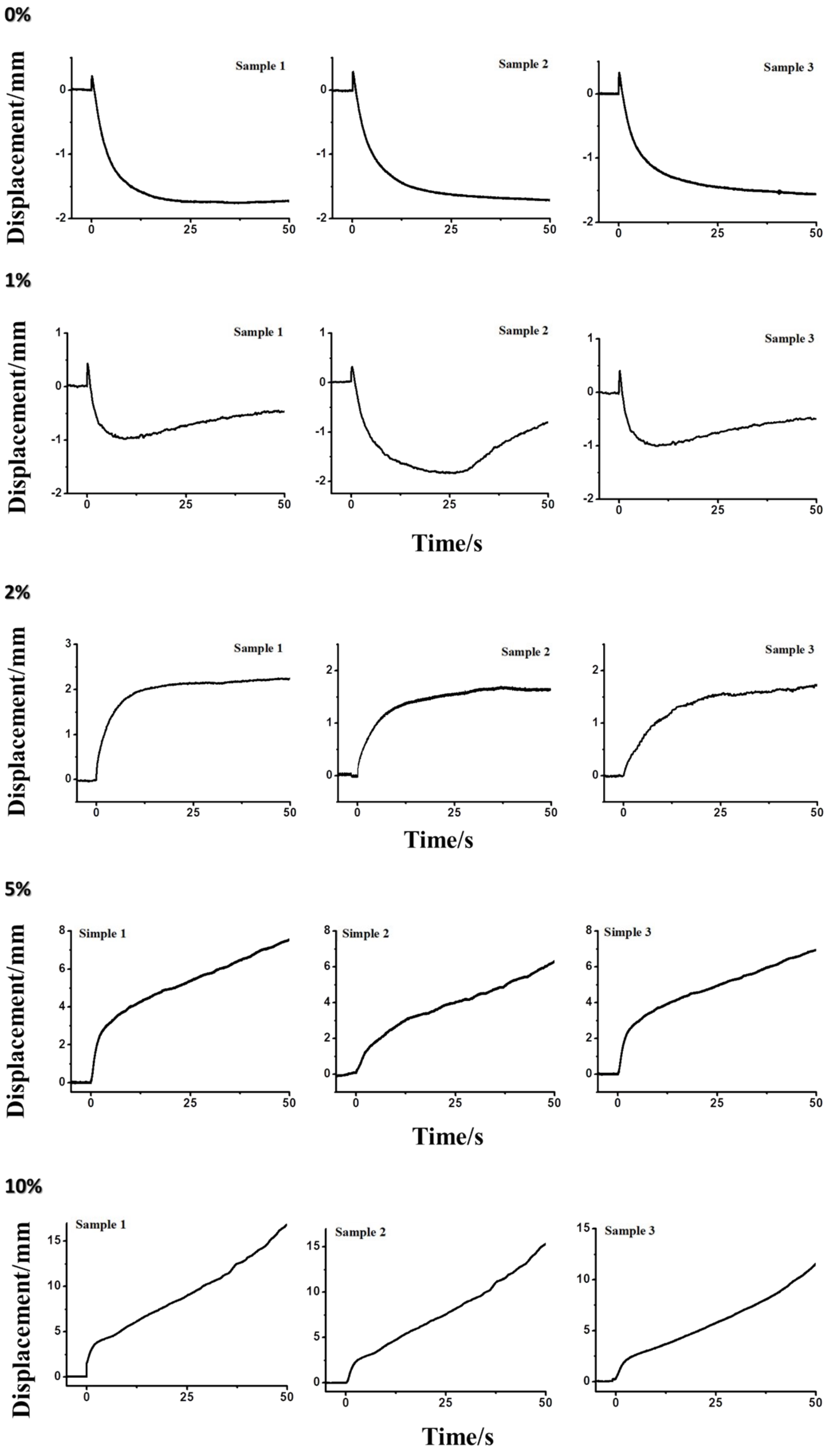

FIG. 3. The measured displacement of three parallel samples of each kind IPMC.

 This journal is © The Royal Society of Chemistry 20xx

5 Fitting effects of the deformations caused by CCNT

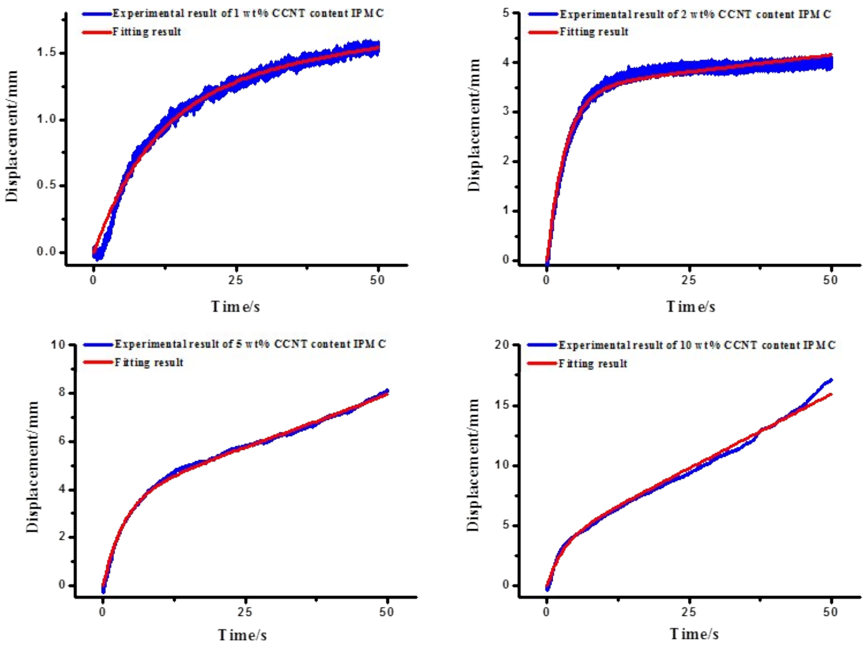

FIG. 4. Fitting effects of the deformations caused by CCNT of various CCNT doped IPMCs.

Table 2. Fitting value and standard error of A, τ and κ of various CCNT doped IPMCs.

CCNT content	1 wt%		2 wt%		5 wt%		10 wt%	
	Value	Standard error	Value	Standard error	Value	Standard error	Value	Standard error
A	1.2730	0.0028	3.4700	0	3.5600	0	3.600	0
τ	10.9393	0.04188	4.1701	0.00443	3.57	0.00466	2.9719	0.01698
κ	0.0056	6.14E-5	0.01383	2.11E-5	0.08756	2.15E-5	0.24633	8.68E-5

Please do not adjust margins
